# The impact of outcome expectancy on therapy outcome in adolescents with borderline personality disorder

**DOI:** 10.1186/s40479-022-00200-1

**Published:** 2022-12-05

**Authors:** Anna-Valeska Bäumer, Lukas Fürer, Carolin Birkenberger, Andrea Wyssen, Martin Steppan, Ronan Zimmermann, Jens Gaab, Michael Kaess, Klaus Schmeck

**Affiliations:** 1 Child and Adolescent Psychiatric Research Department, University Psychiatric Clinics, University of Basel, Wilhelm Klein-Strasse 27, CH-4056 Basel, Switzerland; 2 Division of Clinical Psychology and Psychotherapy, Faculty of Psychology, University of Basel, Basel, Switzerland; 3 University Hospital of Child and Adolescent Psychiatry and Psychotherapy, University of Bern, Bern, Switzerland; 4 Department of Child and Adolescent Psychiatry, Center for Psychosocial Medicine, University Hospital Heidelberg, Heidelberg, Germany

**Keywords:** Borderline personality disorder, Adolescents, Outcome expectancy, Adolescent identity treatment, Dialectical behavior treatment

## Abstract

**Background:**

Outcome expectancy has been found to be a significant predictor of psychotherapy outcome. However, given that severity, chronicity and comorbidity are moderators of outcome expectancy, it is important to provide evidence of whether the same holds true in clinical conditions marked by these attributes, such as in borderline personality disorder (BPD). The aim of the present study was to investigate the role of patients’ outcome expectancy in adolescents undergoing early intervention for BPD using pre-post difference of psychosocial functioning as outcome.

**Methods:**

Forty-four adolescent BPD patients were treated with Dialectical Behavior Therapy for Adolescents (DBT-A) or Adolescent Identity Treatment (AIT). We investigated the effect of outcome expectancy on outcome with type of treatment as moderator. Based on the relevant literature, we assess the correlation between outcome expectancy and pretreatment symptomatology, namely BPD severity, personality functioning, childhood trauma and depression.

**Results:**

The results showed a significant effect of expectancy on outcome (*stand. β =* 0.30, *p =* 0.020) above autoregression. ANOVA analysis revealed no difference between the two treatments. Further, results indicate that pretreatment symptomatology, i.e., depression, childhood trauma and personality functioning dimensions self-direction and intimacy, are associated with early treatment expectancy.

**Conclusion:**

Outcome expectancy as a common factor plays a key role in successful psychotherapy with adolescent BPD patients. Elevated pretreatment depression, childhood trauma and impairment in personality functioning dimensions self-direction and intimacy are risk factors associated with lower expectancy. Low outcome expectancy should be addressed in early psychotherapy to improve the therapeutical process.

**Supplementary Information:**

The online version contains supplementary material available at 10.1186/s40479-022-00200-1.

## Introduction

Understanding mechanism of change in psychotherapy helps to improve psychotherapeutic treatment and treatment outcome [1]. Starting in the 1930s, factors common to diverse methods of psychotherapy have been in the focus of psychotherapy research, showing the relevance of so-called “common factors” such as alliance, empathy, cultural adaptation, therapist differences and expectations [2], which can be differentiated into confidence in therapists as well as in treatments [3].

With regard to the latter, Kirsch [4] has reasoned that a distinction should be made between stimulus expectancies (“occurrence of external events”) and response expectancies (“anticipation of one’s own automatic reactions to various situations and behaviors”). The self-confirming and experience-changing aspects of response expectancy have been shown to be effective in psychotherapy, as well as psychopharmacological and placebo research [5–8]. On a conceptional level, Frank [9] saw psychotherapy as a transformation of meaning, in which providing a plausible rationale for symptoms leads to positive expectancy and thus remoralization. Patients’ expectations can refer to treatment (e.g. role and process expectations) as well as to treatment outcome [10]. Outcome expectancy is defined as patients’ prognostic beliefs about the consequences of participating in treatment [11]. For adults, research indicates that outcome expectancy is positively correlated with outcome [11–14].

Suffering from borderline personality disorder (BPD) causes severe lifetime personal suffering and places a high burden on healthcare systems [15–18]. Specialized early intervention in BPD has the potential to improve global functioning in adolescents with subsyndromal or full-syndrome BPD [19, 20]. For this purpose, several approaches have been adapted for adolescent patients, such as dialectical behavior therapy (DBT-A: [21]), mentalization-based treatment (MBT-A: [22]), schema therapy (SFT-A: [23]) and transference-focused psychotherapy (TFP-A: [24]; AIT: [25]).

According to psychodynamic theory, the inner experience of adolescents with BPD is characterized by identity diffusion that goes along with diffuse, split-off and discontinuous mental representations of the self, others and relationships, distrust and a lack of perspective [26]. From young borderline patients’ point of view, the question arises as to what psychotherapy could hold for them and what they could expect. In understanding the concept of identity diffusion, psychotherapy seems to be a challenge for adolescents with BPD, while crucial interventions are essential regarding the serious consequences of an untreated BPD in adolescence. Wenzel et al. are the only ones to date who have investigated the association between expectation of improvement and therapy outcome in adults with BPD [14]. According to their findings, higher expectations of improvement predict fewer depressive and BPD symptoms after 12 months. To the best of our knowledge, no study has so far analyzed the effect of outcome expectancy on outcome in adolescent BPD patients. As regards other mental disorders in younger age, only a few studies have been conducted. In adolescent populations, expectations of improvement were found to predict outcome in adolescents suffering from depression or obsessive-compulsive disorder [27, 28]. For posttraumatic stress disorder, however, no association with outcome was found [29].

Severity, chronicity and comorbidity of pretreatment mental health characteristics like anxiety, depression and relationship problems are associated with more negative expectancy [30–32].

Depressive symptoms are common in BPD, however, it is discussed that the quality of depressive symptoms is different from patients without BPD [33]. Evidence suggests that certain depressive symptoms such as self-criticism, anger/hostility and hopelessness are elevated in BPD patients [34, 35]. In a heterogeneous sample including personality disorder, higher levels of depression accompanied by hopelessness predicted lower expectancy [36]. In addition, it was found that patients with greater hope reported higher outcome expectations [37, 38]. These results have high face validity considering that in order to build and maintain positive outcome expectancy, a hopeful outlook is pivotal. Insofar, depression coined as hopelessness and outcome expectancy can be considered two highly familiar concepts. Further, Constantino et al. [39] found that severity of Axis II comorbidity is negatively correlated with outcome expectations*.* The authors discuss, as part of these patients’ pathology, bad interpersonal experiences in former treatment as a possible explanation. Many BPD patients did suffer negative, partly traumatic experiences with their important attachment figures and developed insecure attachment and epistemic distrust (defined as a reduced ability to get relevant and generalizable knowledge transmitted in a significant social relationship, as a common origin of rigidity and instability) [40–42]. This in turn may affect the degree to which patients are able to build and maintain outcome expectancies towards psychotherapy, which is interpersonal in nature. However, so far, no studies have shown an association between trauma severity and outcome expectancy.

We studied the association of expectancy with outcome in psychosocial functioning in adolescents with BPD in both a behavioral treatment (DBT-A) and a psychodynamic treatment (Adolescent Identity Treatment, AIT). In accordance with the literature presented above, we hypothesize that high treatment expectancy is associated with better outcome (1). In line with common factor theory, we further hypothesize that the effect of expectancy on outcome is equally strong for both AIT and DBT-A (2). As reviewed, pretreatment symptomatology has proved to be associated with expectancy. We hypothesize that higher pretreatment symptomatology is associated with lower outcome expectancy (3). In accordance with the literature, we consider depression, personality functioning, self-reported childhood trauma and BPD symptomatology. Further, on the basis of results showing the importance of attachment problems in the etiology of personality disorders [40–42] we formulate more specific hypotheses for personality functioning. We hypothesize that the DSM-5 AMPD dimension intimacy shows the biggest association with expectancy (4).

## Method

### Sample

The sample stems from a multicenter nonrandomized controlled trial testing the noninferiority of Adolescent Identity Treatment (AIT: [25]; Basel study center, Switzerland) compared to Dialectical Behavior Therapy for adolescents (DBT-A: [43]; Heidelberg study center, Germany) in adolescent patients with BPD [44]. The trial is registered at clinicaltrials.gov (NCT02518906). Data from both study centers were used. Ethical approval was obtained from the local ethics committees (EKNZ, Ethikkommission Nordwest- und Zentralschweiz, 08–18-20,151, Nr. 2015–230). All adolescents, their parents and the therapists provided written informed consent. Inclusion criteria were: age 13–19 years; three or more BPD criteria in the Structured Clinical Interview for DSM-IV Axis II Personality Disorders (SCID-II: [45]; and identity diffusion according to the Assessment of Identity Development in Adolescence (*t* score > 60; AIDA: [46, 47]). Exclusion criteria were IQ < 80, psychotic disorders, pervasive developmental disorders, severe somatic or neurological disorders, severe and persistent substance addiction, antisocial personality disorder and need for inpatient treatment (for details see Zimmermann et al., 2018). In total, 60 patients fulfilled inclusion and exclusion criteria and were enrolled (AIT: 23, DBT-A: 37). Sixteen patients dropped out (AIT: 6, DBT-A: 10). This resulted in a sample of 44 patients analyzed (AIT: total 17, female 16, male 1; DBT-A: total 27, female 27, male 0; see Table 1 for demographics). We analysed mean differences between completers and dropouts in all variables used in this study. We did not find any significant difference in expectancy and pre-treatment psychosocial functioning, depression, childhood trauma and borderline pathology. We did find a significant difference in pre-treatment personality functioning, where dropouts showed higher impairment (see Supplementary Materials Figs. S3 – S8).

**Table 1:** Demographics, baseline pathology

	AIT	DBT-A	Full Sample	Welch’s Two-Sample t-Test
Gender				
Female	16	27	43	–
Male	1	0	1	–
Age	16.6 (1.5)	15.6 (1.2)	16 (1.4)	t(28.4) = −2.6*,*n* **= 44**
IQ	101.7 (10.1)	105.4 (9.7)	104.1 (9.8)	t(28) = 1.1 (ns), *n* = 42
SCID-II BPD				
Criteria fulfilled	5.6 (1.3)	5.2 (1.4)	5.3 (1.4)	t(36.4) = −0.9 (ns), *n* = 43
3–4 criteria	4	9	13	–
≥ 5 criteria	13	15	28	–
CGAS				
Baseline	48.5 (5.1)	48.6 (8.3)	48.6 (7.2)	t(42) = 0 (ns), *n* = 44
Follow-up	65.6 (9.8)	63.7 (14.8)	64.4 (13)	t(41.8) = −0.5 (ns), *n* = 44
LoPF baseline				
Total score	216.9 (32.8)	236.4 (35.6)	228.9 (35.5)	**t(36.3) = 1.9 (t),*n*** **= 44**
Identity	64.3 (9.8)	67.4 (8.2)	66.2 (8.9)	t(29.9) = 1.1 (ns), *n* = 44
Self-direction	68.3 (14.4)	70.3 (11.4)	69.5 (12.5)	t(28.4) = 0.5 (ns), *n* = 44
Intimacy	48.8 (13.9)	57.4 (13.7)	54.1 (14.3)	**t(33.8) = 2 (t),*n*** **= 44**
Empathy	35.4 (11.4)	41.3 (18)	39 (15.9)	t(42) = 1.3 (ns), *n* = 44
CTQ	48.2 (14.6)	50.6 (18.7)	49.7 (17.2)	t(35.4) = 0.5 (ns), *n* = 42
BDI	36.7 (7.9)	37.5 (10.8)	37.2 (9.8)	t(34.2) = 0.3 (ns), *n* = 41
ZAN	12.5 (3.7)	11.1 (5.3)	11.6 (4.8)	t(41.3) = −1 (ns), *n* = 44
CEQ	38.1 (16)	35.7 (21.6)	36.8 (19.2)	t(35) = −0.4 (ns), *n* = 37

*Abbreviations*: *SCID-II BPD* Borderline criteria in the structured clinical interview for DSM, *CGAS* Children’s Global Assessment Scale, *Follow-up* One-year follow-up, *∆CGAS* CGAS score from one-year follow-up – CGAS score from baseline, *LoPF* Levels of Personality Functioning Questionnaire, *CTQ* Childhood Trauma Questionnaire, *BDI* Beck’s Depression Inventory, *ZAN* Zanarini Rating Scale for Borderline Personality Disorder, *CEQ* Credibility and Expectancy Questionnaire Item 6, *n* Number of observations available; bold = statistically significant and trend-level group differences; significance level: 0–0.001 = ***, 0.001–0.01 = **, 0.01–0.05 = *, 0.05–0.1 = (t), 0.1–1.0 = (ns)

### Dialectical behavior therapy for adolescents (DBT-A)

DBT [48] was developed for the treatment of patients with BPD and chronic suicidal behavior. It has been adapted for adolescents [21]. DBT-A combines strategies from cognitive behavioral therapy (CBT) with acceptance-focused and mindfulness-based principles. The treatment relies on a biosocial etiological model, applies a hierarchy of treatment targets (from life-threatening behavior to quality-of-life interfering behavior) and focuses on the emotional vulnerability and emotional dysregulation of the individual. The patient is validated for his suffering but at the same time is supported in developing a commitment to change. DBT-A is a multiprofessional treatment concept that includes individual therapy and family sessions, as well as a group skills training that covers the modules “mindfulness,” “stress tolerance,” “emotion regulation,” “interpersonal skills,” “self-esteem” and, in contrast to DBT, the additional module “walking the middle path”. There is empirical evidence that DBT-A is superior to enhanced usual care in reducing self-injurious behaviour, suicidality and depressive symptoms [49, 50]. Patients here received 25 individual sessions and 20 sessions of group skills training.

### Adolescent identity treatment (AIT)

AIT [51, 52] is a manualized psychodynamic treatment for adolescents with personality disorders. AIT is administered in 25 sessions and uses adapted techniques of Transference-Focused Therapy (TFP: [53]), namely clarification, confrontation and interpretation, in order to promote identity integration [54]. Therapists emphasize the affect in the here and now and focus on dominant object relation dyads [55]. Further, AIT integrates a behaviorally oriented home plan and an individual amount of family sessions and systemic work with institutions. In a controlled clinical trial, AIT was comparably effective to DBT-A in increasing psychosocial functioning, depressive symptomatology and personality functioning, and reducing BPD symptoms [56]. Therapists were trained in AIT and received weekly supervision from an author of the manual.

### Measures

#### Credibility and expectancy questionnaire (CEQ)

The Credibility and Expectancy Questionnaire is a six-item self-report questionnaire with two factors: credibility (cognitive component) and expectancy (affective component). Expectancy items ask 1) how much patients feel that this treatment will help and 2) by how much they feel their symptoms will improve during therapy. The questionnaire showed high internal consistency (α = .79–.90) and good reliability ([57]; credibility: *r* = .75; expectancy: *r* = .82). We employed the last item (“By the end of the course, how much improvement in your functioning do you really feel will occur?”). The item measures expectancy on an 11-point scale in 10% intervals from 0 to 100%. We use the single item in line with previous research [58, 59]. The item has high face validity and has been shown to be associated with treatment outcome [60]. The CEQ was assessed at different time points with DBT-A and AIT samples. AIT patients filled out the questionnaire after session three, while DBT-A patients filled out the questionnaire at baseline (before therapy began).

#### Childhood trauma questionnaire (CTQ)

The Childhood Trauma Questionnaire (CTQ: [61]) was filled out by patients at baseline. It is a self-report questionnaire for the retrospective assessment of abuse and neglect during childhood and adolescence. Items are rated on a five-point polarized Likert scale and summarized in five dimensions. Here we used the total score, with high values indicating more self-reported abuse and neglect.

#### Beck depression inventory II (BDI)

Depression was assessed using the Beck Depression Inventory II (BDI-II: [62, 63]) at baseline. Here we used the total score. High values indicate more pronounced pathology.

#### Levels of personality functioning (LoPF-Q 12–18)

Personality functioning was assessed at baseline using the Levels of Personality Functioning Questionnaire (LoPF: [64]). The questionnaire consists of 97 items, each with five points. It was developed specifically for the adolescent population. Four dimensions are measured, two of them related to self-functioning, identity and self-direction, and two dimensions related to interpersonal functioning, empathy and intimacy. The questionnaire allows the calculation of a total score indicating personality pathology. High values indicate dysfunction.

#### Zanarini rating scale for borderline personality disorder (ZAN)

The Zanarini Rating Scale for BPD (ZAN: [65]) is an interview assessing BPD pathology during the last week according to the nine DSM-IV criteria. Each criterion is given a value from 0 (no symptoms) to 4 (strong symptoms). Here, we use the total score assessed at baseline, with high values indicating more pronounced borderline symptoms.

#### Children’s global assessment scale (CGAS)

The Children’s Global Assessment Scale (CGAS: [66]) was used to assess outcome in psychosocial functioning. It has been measured both at baseline and 1 year after baseline. Psychosocial functioning is rated on a 100-point scale that includes functioning in different areas (home, school, friends, leisure). The CGAS demonstrated high interrater reliability and concurrent and discriminant validity [67].

#### Ratings

Non-questionnaire ratings (CGAS and ZAN) were conducted by independent raters (research assistants) blind to the course of therapy. However, raters were not blind to the treatment method (AIT, DBT-A), given that the parental study was conducted at two research sites and ratings could not be performed by the same raters for both treatment groups.

### Statistical analysis

We defined outcome in terms of psychosocial functioning (CGAS). The CGAS was measured both before treatment and at 1 year follow-up. In all the following analyses we corrected for the autoregressive effect (pretreatment CGAS).

We considered influential data points using Cook’s distance (*D*, [68]). Cook’s distance assesses the influence of each observation on the effect in a given model [69]. We assessed the influence of all individual observations in a model predicting outcome with expectancy and the autoregressive effect. As a rule of thumb, Cook’s distance is elevated when it is higher than three times the mean distance. Five observations show elevated distance, where all observations lie below .5, except for one observation of the AIT sample, which was high with *D*_*i*_ = 0.86. The respective patient from the AIT group indicated high expectancy (CEQ item score: 80%), being the single maximum expectancy value in the whole data set. However, the patient did only change by three points in psychosocial functioning. This runs against findings presented in the introduction and may be due to an overly compliant answering style. In small samples like here, single observations running against the hypothesized associations may affect results disproportionally. Therefore, the expectancy value of this patient was set to missing. Plots of Cook’s distance before and after setting the observation to missing can be found in the supplementary materials (Figs. S1 & S2).

All analyses are conducted using R [70]. Analyses concerning the association between treatment expectancy and outcome are performed using lavaan [71] with a full information maximum likelihood estimator (FIML). The variables were standardized before analysis, resulting in standardized coefficients being reported.

**Fig. 1:**
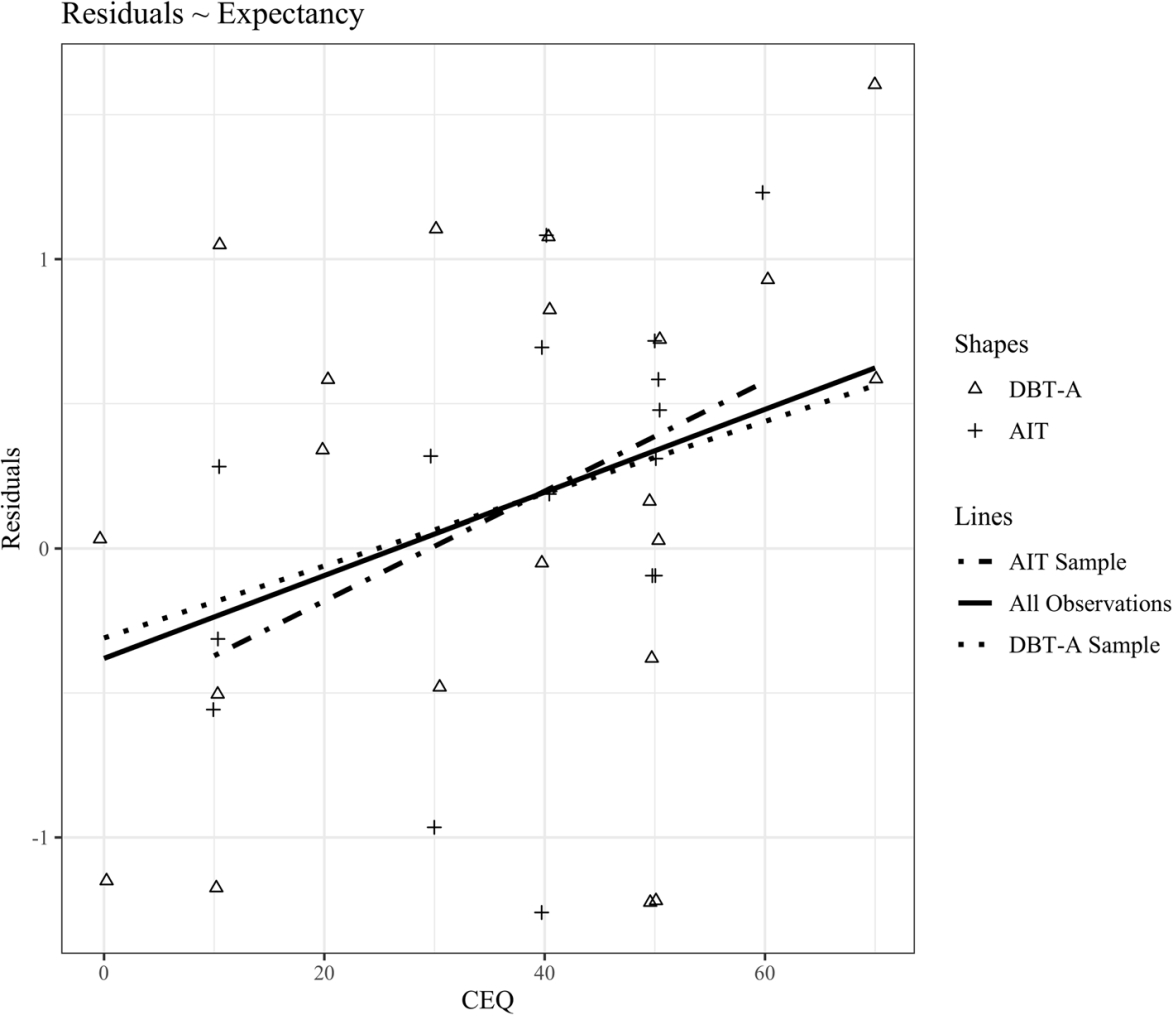
Effect of Expectancy on Outcome Residuals. Abbreviations: Residuals = Residuals from a linear autoregressive model predicting follow-up CGAS using pretreatment CGAS. CEQ = Credibility and Expectancy Questionnaire (CEQ) Item 6; DBT-A = Dialectical Behavior Therapy; AIT = Adolescent Identity Treatment; Point shapes indicate groups; Least square lines are drawn for the overall sample (all observations) and for each treatment individually

The main hypothesis concerns the association between expectancy and outcome. It was tested in a global model, including observations from both treatments. In this global model, outcome was predicted by expectancy and the autoregressive effect. In order to test whether the associations differ across the two treatments, we fitted the same model with treatment as grouping variable in a restricted and unrestricted version. For the expectancy predictor, the restricted model used treatment-wise random intercept and a fixed slope for both treatments. The unrestricted model allowed for both treatment-wise random intercept and random slope. In both the unrestricted and the restricted model, the autoregressive effect was fixed with a treatment-wise random intercept and fixed slope. Model fits are compared using ANOVA. A better fit of the unrestricted model would indicate a stronger association between expectancy and outcome in one treatment when considering the autoregressive effect. As a graphical representation of the effect (see Fig. 1), we calculated the residuals from the linear autoregressive model (outcome in the CGAS explained by pre-treatment CGAS). We then plotted the expectancy values against the residuals and added least squares lines for the overall sample and the individual treatments.

Our secondary hypotheses concern the association between treatment expectancy and pretreatment symptomatology. We hypothesized that pretreatment symptomatology is associated with expectancy. Our hypotheses are mainly deducted from literature; however, the number of studies is still underwhelming, and this is the first manuscript to study outcome expectancy in adolescents with BPD. Further we are also limited by the sample size. Given the rather inductive nature of these hypotheses we decided to refrain from path analyses and report the correlation matrix of the pretreatment symptomatology variables and expectancy.

## Results

### Demographics and baseline pathology

Table 1 summarizes total and group-wise demographics and instrument scores in the sample used. Due to missing values, not all analyses could be conducted on the whole sample. The number of available observations is indicated with each measure (*n*). Given that our analyses use the combined DBT-A and AIT samples, we also provided t-statistics to ensure homogeneity in the variables used over the samples. A significant group difference was found for age, with the mean age in the AIT sample being 1 year older than in the DBT-A sample.

### Expectancy and outcome

In a global model we predicted outcome in the CGAS with a multiple regression model using expectancy and autoregression as predictors over the whole data set. Results indicated a significant effect of expectancy on outcome (*stand. β =* 0.30, *p =* 0.020) above autoregression (*stand. β =* 0.49, *p* < 0.001). In order to test whether the effect differs across treatments, model fits of an unrestricted (random slope per treatment for expectancy) and a restricted (fixed effect per treatment for expectancy) model are compared (see statistical analysis). ANOVA indicates no significant increase in fit for the unrestricted model (*chi-squared diff.* = 0.48, *p =* 0.489). A scatterplot of expectancy values against the residuals from the linear autoregressive model with least square lines is shown in Fig. 1.

### Pretreatment symptomatology and outcome expectancy

Individual correlations are presented in Table 2. We find that higher self-reported childhood trauma (*r* (33) = −.36, *p* = 0.031), higher depression (*r* (32) = −.38, *p* = 0.024), and higher impairment in personality functioning (*r* (35) = −.31, *p* = 0.062), specifically dimensions self-direction (*r* (35) = −.36, *p* = 0.027) and intimacy (*r* (35) = −.28, *p* = 0.093), are associated with lower outcome expectancy.

**Table 2:** Correlation Matrix Pretreatment Symptomatology & Outcome Expectancy

Variable	1	2	3	4	5	6	7	8
1. CEQ	–							
2. CTQ	− 0.36* [− 0.62, − 0.04]							
3. BDI	−0.38* [− 0.64, − 0.05]	0.29(t) [− 0.02, 0.55]						
4. LoPF Total Score	**−0.31(t)** [− 0.58, 0.02]	0.34* [0.04, 0.58]	0.54*** [0.28, 0.73]					
5. LoPF Identity	−0.18 [− 0.47, 0.16]	0.23 [− 0.08, 0.5]	0.53*** [0.27, 0.72]	0.72*** [0.53, 0.84]				
6. LoPF Self-Direction	−0.36* [− 0.62, − 0.04]	**0.27(t)** [− 0.04, 0.53]	0.64*** [0.41, 0.79]	0.74*** [0.57, 0.85]	0.54*** [0.29, 0.72]			
7. LoPF Empathy	−0.04 [− 0.36, 0.28]	0.04 [− 0.27, 0.34]	− 0.13 [− 0.42, 0.18]	0.55*** [0.3, 0.73]	0.15 [− 0.15, 0.43]	0.05 [− 0.25, 0.34]		
8. LoPF Intimacy	**− 0.28(t)** [− 0.55, 0.05]	0.41** [0.12, 0.64]	0.6*** [0.36, 0.76]	0.78*** [0.63, 0.87]	0.51*** [0.25, 0.7]	0.57*** [0.33, 0.74]	0.11 [−0.2, 0.39]	
9. ZAN	−0.2 [− 0.49, 0.14]	0.24 [− 0.06, 0.51]	**0.27(t)** [− 0.04, 0.54]	0.11 [− 0.19, 0.4]	0.08 [− 0.22, 0.37]	0.15 [− 0.16, 0.43]	−0.05 [− 0.34, 0.25]	0.16 [− 0.14, 0.44]

*CEQ* Credibility and Expectancy Questionnaire Item 6, *CTQ* Childhood Trauma Questionnaire, *BDI* Beck’s Depression Inventory, *LoPF* Levels of Personality Functioning Questionnaire, *ZAN* Zanarini Rating Scale for Borderline Personality Disorder; bold = statistically significant and trend-level associations; significance level: 0–0.001 = ***, 0.001–0.01 = **, 0.01–0.05 = *, 0.05–0.1 = (t), 0.1–1.0 = (ns), [] = 95% confidence intervals

## Discussion

In this manuscript we have studied the effect of outcome expectancy on outcome in two treatments for adolescents with borderline personality disorder. Further, we have tested the association between outcome expectancy and BPD severity, personality functioning, childhood trauma and depression. Our results confirmed the first hypothesis of a significant positive correlation between treatment expectancy and treatment outcome in adolescent BPD patients, with higher outcome expectations in early treatment being associated with higher psychosocial functioning at follow-up. In accordance with current common factor theory research, there was no significant difference between the DBT-A and AIT group [12, 56].

As derived from literature, the pretreatment variables childhood trauma, depression, personality functioning total score (trend level) and the personality functioning dimension intimacy (trend level) were found to be associated with outcome expectancy. This is in line with previous findings showing that severity of borderline symptomatology as well as a higher burden of global symptomatology are associated with lower expectancy [30–32]. As in previous studies, higher baseline ratings in depression assessed with BDI-II were also associated with lower outcome expectations. Looking pessimistically into the future, low self-confidence and poor self-esteem could contribute to negative outcome expectations [27, 36].

With respect to BPD, it is possible that interpersonal difficulties and instability in relationships in BPD reduce a positive outcome expectation of psychotherapy as an interpersonal treatment method. Chronic emptiness, affective instability or serious identity disturbance could also negatively affect outcome expectations. According to Kirsch, a vicious circle of negative expectations is found in depression and anxiety disorder [6, 72]. Likewise, a vicious circle of negative expectations might be perpetuated by the typical instability in self-perception, in relationships and in affectivity seen in adolescent patients with BPD.

Specifically, our results indicated that impairment of intimacy, self-direction as well as self-reported childhood trauma are significantly associated with lower outcome expectancy. It is plausible that these experiences lead to low epistemic trust among the patients, as patients with BPD have been found to expect few benefits from their treating psychotherapist [73] and to have deficits in epistemic trust [74]. As BPD patients are known to be at risk of discontinuing treatment, epistemic trust is discussed as an early marker to identify who may respond successfully to different therapeutical interventions [75]. In their developmental model of BPD, Fonagy et al. conceptualize impairment in epistemic trust, defined as a reduced ability to get relevant and generalizable knowledge transmitted in a significant social relationship, as a common origin of rigidity and instability [76]. In an adaption of the model for adolescent BPD patients, Bo et al. emphasize a reduction of hypermentalizing and epistemic mistrust as principal therapeutical targets in order to establish a mutual process in which the capacity to learn from each other could flourish [77]. Reopening of epistemic trust could be attained through the therapeutical qualities of consistency, coherence and continuity [78].

In particular, the optimistic stance (“holding the hope for the patient”) in AIT [25] as well as dialectical interventions and formulating common goals in DBT-A are essential key features reflecting therapists’ hope and positive outcome expectancy. We did not hypothesize to find the strong association between self-direction (self-congruence and purposefulness) and outcome expectancy (cf. [79]), the result however has high face-validity from a clinical perspective. A higher degree of self-direction, in terms of the ability to formulate goals and pursue them, may foster patients’ engagement and responsibility in terms of positive change in the therapeutic process. In association with the concept of psychological mindedness, a higher capacity for self-reflection and for recognizing psychological processes and meanings could contribute to positive outcome expectancy [80, 81].

### Strengths and limitations

Several strengths and limitations of our study must be addressed. To the best of our knowledge, it is the first study to focus on outcome expectancy in adolescent BPD patients. This study not only investigates the association between outcome expectancy and treatment outcome among young BPD patients, but also, covariates in pretreatment symptomatology of outcome expectancy are identified. To confirm the (preliminary) results, a replication in a bigger and more gender balanced sample is needed. However, our findings are largely consistent with the reviewed expectancy literature. Despite the small sample, significant and trend level associations are found. The correlations between pretreatment symptomatology and outcome expectancy are hardly clear evidence of causality. Given a bigger sample, future research should focus on to what extent outcome expectancy mediates the effect of pretreatment symptoms on outcome. However, our results suggest that outcome expectancy is a relevant factor in successful psychotherapy with young BPD patients and that higher pretreatment symptomatology is a risk factor for the development and maintenance of good outcome expectancy. A major limitation of the study is that outcome expectancy was assessed at different time points in the two samples (AIT after session 3, DBT-A before treatment). It was impossible to overcome this limitation. However, we found no statistical difference in the extent of the effect of outcome expectancy on outcome across treatments. Further investigations are also needed to clarify the role of outcome expectancy as a state-like, dynamic factor during the course of treatment [79, 82–84]. According to Constantino et al., limitations in expectancy assessment seem to be a general problem in expectancy research [12].

### Clinical implications

The results of this study have several therapeutical implications for psychotherapy with young BPD patients. Therapists should address patients’ outcome expectations early in psychotherapy and foster positive expectations [85]. In particular, early interventions modifying outcome expectancy could benefit patients with higher baseline ratings in depression, impairment in personality functioning or higher self-reported childhood trauma. Identifying and working on negative expectancy will improve the psychotherapeutic process in this special group of patients [14].

Assuming personality pathology, mentalizing capacity and personality functioning as related concepts, a significant relationship between severity of borderline symptomatology and interpersonal functioning mediated by mentalizing capacity was found [86]. Early intervention in patients with low treatment expectancy might focus on mentalization capacity to improve interpersonal functioning, which could increase treatment expectancy. In patients with persistent low expectancy this lack of confidence in the therapist and / or the treatment approach should become the first focus of psychotherapy.

Bearing in mind the respect for patients’ autonomy and right to self-determination, it is important to give adequate and truthful information about the nature of psychotherapy and, as a moral obligation, about the effect of common factors such as expectancy [87–89]. In regard to patients’ cultural background, therapists could adjust the therapeutic rationale to enhance positive outcome expectancy [90, 91]. Positive outcome expectancy could finally result in higher engagement in psychotherapy [92]. Working with outcome expectancy could be finally understood as validation of the patient’s sense of agency [78]. In conclusion, by understanding psychotherapy as an interpersonal endeavor, outcome expectancy could play a key role in successful treatment with young BPD patients.

## Conclusion

This study indicates the importance of outcome expectancy in successful psychotherapy with young BPD patients. Especially in patients with low expectancy early modifying interventions should be considered. Particular attention should be paid to patients with high self-reported childhood trauma, depression and impaired personality functioning who are at risk for low expectancy. More research is needed to confirm our results and study relevant mechanisms of change in outcome expectancy.

## Supplementary Information

**Additional file 1: Fig. S1.** Cook’s Distance before Exclusion of Data Point. **Fig. S2.** Cook’s Distance after Exclusion of Data Point. **Fig. S3.** Comparison of Means in CEQ Dropouts vs. Completers. **Fig. S4.** Comparison of Means in CGAS Dropouts vs. Completers. **Fig. S5.** Comparison of Means in BDI Dropouts vs. Completers. **Fig. S6.** Comparison of Means in CTQ Dropouts vs. Completers. **Fig. S7.** Comparison of Means in LoPF Dropouts vs. Completers. **Fig. S8.** Comparison of Means in ZAN Dropouts vs. Completers.

## Data Availability

The datasets generated and/or analysed during the current study are not publicly available due to ongoing analyses of data produced by the clinical trial but are available from the corresponding author on reasonable request.
