# Supplementary material for: The impact of outcome expectancy on therapy outcome in adolescents with borderline personality disorder
Source: Borderline Personal Disord Emot Dysregul. 2022 Dec 5;9:30. doi: 10.1186/s40479-022-00200-1 (PMC9721041; doi:10.1186/s40479-022-00200-1)
Supplement: Supplementary file 1 — Additional file 1: Fig. S1. Cook’s Distance before Exclusion of Data Point. Fig. S2. Cook’s Distance after Exclusion of Data Point. Fig. S3. Comparison of Means in CEQ Dropouts vs. Completers. Fig. S4. Comparison of Means in CGAS Dropouts vs. Completers. Fig. S5. Comparison of Means in BDI Dropouts vs. Completers. Fig. S6. Comparison of Means in CTQ Dropouts vs. Completers. Fig. S7. Comparison of Means in LoPF Dropouts vs. Completers. Fig. S8. Comparison of Means in ZAN Dropouts vs. Completers. [file 40479_2022_200_MOESM1_ESM.docx]

**Figure S1**

*Cook’s Distance before Exclusion of Data Point*

**Figure S2**

*Cook’s Distance after Exclusion of Data Point*

**Figure S3**

*Comparison of Means in CEQ Dropouts vs. Completers*

Comparison of Means for CEQ in Dropouts vs. Completers, Welch Two Sample T-Test *t*(16.7) = 0.7, *p =* 0.493; CEQ = Credibility and Expectancy Questionnaire (CEQ) Item 6; Dropout 0 = completers, 1 = Dropouts.

**Figure S4**

*Comparison of Means in CGAS Dropouts vs. Completers*

**

Comparison of Means for CGAS in Dropouts vs. Completers, Welch Two Sample T-Test: *t*(27.99) = -1.43 , *p* = 0.16; CGAS = Children’s Global Assessment Scale; Dropout 0 = completers, 1 = Dropouts.

**Figure S5**

*Comparison of Means in BDI Dropouts vs. Completers*

**

Comparison of Means for BDI in Dropouts vs. Completers, Welch Two Sample T-Test: *t*(11) = 0.67 , *p* = 0.52; BDI = Beck’s Depression Inventory; Dropout 0 = completers, 1 = Dropouts.

**Figure S6**

*Comparison of Means in CTQ Dropouts vs. Completers*

**

Comparison of Means for CTQ in Dropouts vs. Completers, Welch Two Sample T-Test: *t*(25.63) = -0.49 , *p* = 0.63; CTQ = Childhood Trauma Questionnaire; Dropout 0 = completers, 1 = Dropouts.

**Figure S7**

*Comparison of Means in LoPF Dropouts vs. Completers*

**

Comparison of Means for LoPF in Dropouts vs. Completers, Welch Two Sample T-Test: *t*(24.91) = 2.76 , *p* = 0.01; LoPF = Levels of Personality Functioning Questionnaire; Dropout 0 = completers, 1 = Dropouts.

**Figure S8**

*Comparison of Means in ZAN Dropouts vs. Completers*

**

Comparison of Means for ZAN in Dropouts vs. Completers, Welch Two Sample T-Test: *t*(18.64) = 1.42 , *p* = 0.17; ZAN = Zanarini Rating Scale for Borderline Personality Disorder; Dropout 0 = completers, 1 = Dropouts.
